# Analysis of Interlimb Asymmetry in Patients Undergoing Simultaneous Bilateral Total Knee Arthroplasty

**DOI:** 10.1371/journal.pone.0129783

**Published:** 2015-06-24

**Authors:** Yu Yang, Gong Long, Wang Zhenhu

**Affiliations:** Department of orthopedic, 252 Hospital of Chinese PLA, Baoding City, Hebei, China; University of Rome Foro Italico, ITALY

## Abstract

Simultaneous bilateral TKA (SBTKA) has been a favored surgical solution due to reduced costs and patient suffering. The purpose of the present study was to investigate the risk factors of asymmetric recovery in patients who underwent SBTKA and whether that affected quality of life. A total of 187 patients undergoing SBTKA were included. During this study, patients underwent physical examination (knee swelling, active range of motion (ROM) of knee and quadriceps strength) and completed three surveys (VAS pain rating, Short Form-36 and requisite information lists in this study). Our results reveal interlimb asymmetries existed at least two years postoperatively. Between-limb differences in active ROM, quadriceps strength, and VAS pain scores were significantly detected in our study. Risk factors included being female, being older, and having high BMI and high levels of anxiety and depression; different diagnosis and different component size could be risk factors. Finally, interlimb differences in VAS pain scores and active ROM were negatively associated with SF-36 scores. However, interlimb differences in swelling and quadriceps strength were unrelated to SF-36 scores. Risk factors of asymmetric recovery should be evaluated and appreciated due to their significant impact on patients’ quality of life. Before performing SBTKA, clinicians should consider possible risk factors and inform patients of asymmetric recovery between limbs, which could help decrease the unnecessary consultations and postoperative patient dissatisfaction.

## Introduction

Simultaneous bilateral total knee arthroplasty (SBTKA) has been favored due to lower costs and less surgical trauma and complications compared to staged bilateral TKA [1,2],and compared to the same duration of hospital stay and recovery time as unilateral TKA [1,3–6].Although the results of SBTKA are well substantiated, there is a dearth of data reflecting the difference in recovery between sides, that is, interlimb asymmetry. However, interlimb asymmetry is clinically important as it has been associated with negative consequences such as increased medical expense, impaired satisfaction, and decreased bone mass density in the more symptomatic limb [7]. Additionally, patients whose knees showed significantly asymmetrical performance after SBTKA always require more persistence and time to recovery [1, 7, 8].Patients desire to achieve the same feeling between limbs, especially those with high levels of depression and anxiety [9]. Therefore, it is necessary to identify high-risk patients prior to SBTKA to avoid those problems.

To the best of our knowledge, almost all of the published studies related to asymmetry in TKA have been based on participants who accepted unilateral or staged bilateral TKA [10–15].Despite a few studies that described the asymmetry between limbs among individuals undergoing simultaneous bilateral TKA, these studies have focused on interlimb difference in the gait and component size[16–19]. Few studies to date have investigated the difference between limbs in clinical results such as pain scores, active range of motion, and quadriceps strength, factors to which we attach great importance in clinical practice. Therefore, the primary purpose of this study was to investigate interlimb differences in clinical results among SBTKA patients. Also we examined the potential risk factors of interlimb asymmetry and its effects on postoperative quality of life. It was hypothesized that there existed significant interlimb differences in clinical results and potential risk factors. In addition, we hypothesized that interlimb asymmetry would negatively affect patients’ quality of life.

## Materials and Methods

### Study population

Through a defined time period, all consecutive patients with bilaterally symptomatic osteoarthritis were enrolled in this prospective study. Patients signed written consent before the first assessment, which contained information on risk, complications, and quadriceps strength as measured by a machine as well as necessary clinical visit follow-up. The study was approved by the ethics committee of 252 hospital of Chinese PLA (No. Ortho-22) and was carried out in accordance with the Declaration of Helsinki. This patient was excluded if he or she (1) had high risks for SBTKA following the rigorous selection, (2) had had obvious asymmetry in appearance, quadriceps strength, pain, knee girth or active ROM prior to surgery (difference greater than 20%, see“pre-, peri-, and postoperative evaluation”below), (3) had postoperative infection in one or both knees, (4) was unable to complete our survey independently, (5)accepted different types of components,(6) had been diagnosed with deep vein thrombosis(DVT), pulmonary embolism, or severe arteriosclerosis obliterans in one or both limbs, or (7) had undergone joint preservation surgery or total joint arthroplasty. A minimum of two-year follow-up was performed.

### Surgical procedure

We administered all patients with prophylactic antibiotics (vancomycin or cefotaxime) before the skin incision routinely and then every 8 hours for 24 hours. Epidural anesthesia was used in SBTKA procedure. All operations were performed by the same surgical team. Cemented cruciate-retaining prosthesis (Gemini MK-II, Link, Germany) with patellar resurfacing was used for all patients in this study. The approach to expose the capsule was median incision. The osteotomy of femur and tibia were intra or extra-medullary guided fashion, respectively. Recollected blood was filtered and washed in the recovery room and then retransfused into the patient within 6 hours. The drainage tube was promptly extracted at postoperative 24h. The pneumatic tourniquet was sequentially applied to both sides at 300mm Hg. Both were used from the beginning of femur osteotomy to the end of tibia osteotomy and then released following closure of the joint capsule. Patients received a patient-controlled analgesic (PCA) pump during postoperative 48 hours. For relieving postoperative pain, all patients received routinely diclofenac sodium (50 mg, tid) orally during their hospital stay and tramadol (100 mg, tid) after discharge. Preventive anticoagulant therapy (10mg rivaroxaban every day) began within 12 hours after operation and continued for 14 days.

### Pre-, peri,- and postoperative evaluation

Active ROM is of importance to postoperative functional training and recovery following TKA; it is an objective indicator of recovery progress. Therefore, we selected it as the primary clinical outcome to describe the characteristics of interlimb asymmetry and its potential determinants.

Patients were seated in an electromechanical dynamometer (KinCom; Chattecx, Harrison, TN) to measure maximum quadriceps strength (Newton, N) with both knees flexed at 75°. They were asked to extend their knee as much as possible for 3 s. Two attempts of maximal contraction were performed and the greater one was recorded. The quadriceps strength of both limbs was measured and recorded. Patients sat with their hands on their laps on a chair that was designed to stabilize the body and minimize synkinetic movements.

A Lafayette Gollehon extendable goniometer was applied to measure active ROM. Patients in supine position were asked to slide their heels toward the buttocks and to flex their knees maximally. Then knee ROM was recorded.

In addition to those above, we recorded swelling and pain scores. Pain intensity was evaluated by a 10cm visual analogue scale (VAS) in which 0 represented no pain and 10 represented the worst imaginable pain intensity.

Between-limb difference greater than 20% in active ROM was defined as asymmetry in this study. Assessments of these four parameters were performed at seven time points (preoperatively, and postoperatively at 1, 4, 12, 24, 52, and 104 weeks).

SF-36 survey was recorded at 12 months postoperatively to measure patients’s quality of life. Before discharge, we routinely performed radiograph evaluations and then excluded operation-related factors that produced interlimb asymmetry.

### Risk factors construct

We listed possible risk factors to construct a survey questionnaire to the best of our best knowledge. It consisted of basic demographic information,diagnosis, physical examination, comorbid conditions, psychological factors, and operational details. Psychological factors included anxiety and depression, which were measured by Hospital Anxiety and Depression Scale (HADS) (dichotomized < 8 and ≥ 8). This questionnaire was completed by the patients prior to surgery.

### Statistical analysis

We analyzed interlimb asymmetry through comparing the dominant and non- dominant side. Specifically, the dominant side was defined by better performance in active ROM. Between-limb asymmetry was examined using a paired t-test. In addition, we examined the linear relationship between patients’ magnitude of asymmetry and scores on the SF-36 using a Pearson correlation. Multiple logistic regression analysis was used to determine the risk factors for asymmetry in pain. Two-way ANOVAs were used to detect whether a significant relationship existed between time intervals and gender, BMI, and other variables. Data are shown as mean ± SD or as percentages. It was assumed that all these statistics were normally distributed. Statistical significance was set at p-value ≤0.05. Statistics analyses were performed by SAS Statistical Software 9.1.3.

## Results

From May 1, 2013 to October 1, 2014, a total of 250 participants underwent SBTKA and 63 cases were excluded. Particularly, 32 patients had been considered as interlimb asymmetry before surgery by our definition; 4 patients had infection in one knee; 8 patients were unable to complete our survey due to cerebral stroke or mental disorder; 7 patients accepted different TKA’components; 2 patients were diagnosed with symptomatic DVT; 4 patients showed severe arteriosclerosis obliterans in one limb; and 6 patients were missing during follow-up. Ultimately, 187 patients were analyzed in this study. Preoperative and operative details are shown in Table 1. There was no significant interlimb difference in lower limb alignment based on X-rays.

**Table 1:** Demographic characteristics and baseline information.

Variable	DS	NS	P value
Age (years)	64.8±5.3		-
Sex (male/female)	56/131		-
Height (m)	1.73±1.0		
Mass (kg)	85.3±5.4		
BMI (kg/m2)	28.5±3.4		-
Quadriceps strength(N)	252.1±48.1	260.2±52.5	0.1230
ROM (°)	88.2±12.8	85.8±14.5	0.0906
VAS pain scores	4.82±1.32	4.63±1.53	0.1561
Knee girth (cm)	40.21±2.61	39.87±2.52	0.2008

DS Dominated Side, US non-dominated Side, ROM range of motion, VAS visual analogue scale

P value significant at 0.05.

### Active range of motion (ROM), quadriceps strength, pain, and swelling between limbs

There was no significant difference between limbs in quadriceps strength, pain, active range of motion (ROM), and swelling prior to SBTKA. However, there were significant differences in quadriceps strength, VAS pain scores, and active ROM between limbs during follow-up (Table 2). Notably, significant difference in knee girth failed to be detected in the present study (Table 2).

**Table 2:** Clinical outcomes of two sides after SBTKA.

Variable	Active ROM (°)			Quadriceps strength(N)			VAS pain scores			Knee Girth (cm)		
	DS	NS	P value	DS	NS	P value	DS	NS	P value	DS	NS	P value
Before surgery	88.2±12.8	85.8±14.5	0.0906	260.2±52.5	252.1±48.1	0.1230	4.63±1.53	4.82±1.32	0.1561	40.21±2.61	39.87±2.52	0.2008
1 week	95.5±11.6	91.2±12.3	0.0006*	90.4±20.3	82.3±23.6	0.0004*	3.52±1.72	3.90±1.53	0.0246*	45.62±2.72	45.1±2.82	0.0703
4 weeks	103.5±13.5	98.5±14.2	0.0005*	165.4±39.8	146.2±42.1	0.0305*	2.89±1.00	3.35±1.68	0.0097*	44.13±2.55	43.83±2.73	0.2728
12 weeks	106.8±10.1	101.2±9.8	<0.0001*	306.8±41	276.4±45.2	0.0041*	2.24±1.82	2.78±1.78	0.0039*	42.04±2.43	41.67±2.64	0.1593
24 weeks	108.3±7.8	101.8±8.5	<0.0001*	323.4±47.8	302.6±45.2	<0.0001*	1.91±1.42	2.42±1.33	0.0004*	41.13±2.8	41.00±2.62	0.6432
52 weeks	113.2±8.2	104.5±10.6	<0.0001*	387.8±46.2	370 ±44.3	0.0002*	1.63±1.04	2.1±.12	<0.0001*	40.85±2.51	40.45±2.63	0.1333
104 weeks	114.5±7.9	107.8±9.8	<0.0001*	396.5±44.5	382.5±41.2	0.0017*	1.15±0.98	1.73±1.10	<0.0001*	40.20±2.44	40.00±2.58	0.4417

DS Dominated Side, US non-dominated Side

P value significant at 0.05.

* there was a statically significant difference

### Risk factors of inter-limb asymmetry

Table 3 shows risk factors associated with the SBTKA procedure. According to our criterion of asymmetry (difference in active ROM >20%), possible risk factors presented were female, aged, high BMI, high levels of anxiety and depression, and different diagnosis and different component size in the adjusted multivariate logistic regression analysis (Table 3).

**Table 3:** Risk factors for inter-limb asymmetry.

Risk factors	Unadjusted			Adjusted ^a^		
	OR	95% CI	P value	OR	95% CI	P value
Female (vs Male)	1.23	0.78–1.68	0.0038*	-	-	-
Age (5 years)	1.62	1.13–2.11	0.0098*	-	-	-
BMI > 25 kg/m2	1.76	1.18–2.34	0.0032*	1.68	1.36–2.26	0.0214*
Hypertension	1.10	0.82–1.38	0.0623	1.02	0.74–1.30	0.2312
Diabetes mellitus	1.53	1.18–1.88	0.0325*	1.45	1.10–1.80	0.0531
Current smoking	1.40	0.98–1.82	0.0135*	1.35	0.93–1.77	0.1214
Lower extremity vascular disease	1.58	1.02–2.14	0.0431*	1.48	0.92–1.94	0.2352
High anxiety and depression	2.36	1.82–2.90	0.0003*	2.21	1.67–2.75	0.0022*
Different diagnosis	1.98	1.32–2.64	0.0021*	1.90	1.24–2.56	0.0353*
Different component size	1.36	0.81–1.91	0.0231*	1.28	0.73–1.82	0.0392*

BMI Body mass index, OR odds ratio

P value significant at 0.05.

^a^ Adjusted for age and gender

* there was a statically significant difference

### Relationship between the level of asymmetry and Short-Form 36

Interlimb differences in VAS pain scores and active ROM were significantly negatively associated with SF-36 scores (Table 4). However, interlimb differences in swelling and quadriceps strength were unrelated to SF-36 scores (Table 4). A two-way ANOVA was performed on time interval and gender/BMI: (1) the interaction of asymmetry between limbs was significant (P = 0.0032), (2) time interval had significant effects on asymmetry between limbs (P = 0.0022),and (3) gender/BMI over asymmetry between limbs were not significant (P = 0.0055/0.0213).

**Table 4:** Relationship between the magnitude of asymmetry and Short-Form 36

Short Form 36	Difference in clinical outcomes			
	Quadriceps strength	VAS pain scores	Active ROM	Swelling
r	-0.492	-0.745	-0.722	-0.313
P value	0.1531	0.0318*	0.0012*	0.2162

ROM range of motion, VAS visual analogue scale

P value significant at 0.05.

* there was a statically significant difference

## Discussion

Our results suggest that there is significant asymmetric recovery between limbs for SBTKA patients just as there is with staged bilateral TKA and unilateral TKA. The present study provides novel evidence that interlimb difference in clinical outcomes after SBTKA appear early after surgery and may extend for two years postoperatively or longer. Interlimb asymmetry can be detected mainly by using the measures of active ROM, pain level, and quadriceps strength.The level of interlimb differences in VAS pain score and ROM were negatively associated with scores on the SF-36. Finally, in the adjusted multivariate logistic regression analysis, we observed that age, female, high BMI, high anxiety and depression, different diagnosis, and different component size were significant risk factors.We offer these possible explanations:

1. Patient-reported questionnaires aim to assess pain, quality of life, and function in activities of daily living. Studies have found that psychological factors greatly affect the results of these questionnaires [20, 21]. For example, catastrophizing, which is a negative cognitive and affective response to pain, magnifies the difference in pain-related symptoms, and increases rumination and focus on pain with excessive attention[20, 22], all of which can influence pain-related outcomes. Further, catastrophizing is rather common among aged, female, and depressed patients [21–23]. Therefore, just as we observed, there were higher risks of experiencing significant asymmetrical pain between limbs among aged, female, and depressed individuals. Studies have indicated that pain level directly affects active ROM and quadriceps strength [22, 23]. Specifically, the less painful limb exhibits more exercise with the result of better active ROM and quadriceps strength, while more pain leads to increased muscle spasm and compromised quadriceps strength [23]. In other words, the experience of asymmetrical pain can lead to asymmetrical active ROM and quadriceps strength.
2. Asymmetrical load distribution in a lower extremity after surgery has been revealed to broaden the gap between limbs and increase the risk of asymmetrical recovery progress [16]. However, high BMI can aggravate such an asymmetrical load distribution [14, 16].Therefore, patients with high BMI were at larger risk for interlimb asymmetry compared with low BMI.
3. Although in most cases both knees are diagnosed with degenerative osteoarthritis, there is a possibility that the specific etiology of each knee is different. For instance, one side with traumatic osteoarthritis may indirectly lead to secondary osteoarthritis in the contralateral side due to uneven distribution of weight load. Different etiology has different pathophysiological process [24], which could affect recovery after SBTKA.
4. Although bilateral osteoarthritis was usually symmetric in deformity, it is difficult to guarantee the same component size applied in bilateral TKA. Brown reported that the prevalence of femoral component asymmetry was 6.7–9.2% and tibial component asymmetry was 0–8.7% among SBTKA procedures [17]. Components in articular cavity can cause foreign body reaction, based on component size and material [24]. Therefore, different component size could increase the risk of interlimb asymmetrical recovery after surgery.

In addition, we investigated the effect of interlimb difference after SBTKA on quality of life.Specifically, between-limb differences in pain scores and active ROM were negatively associated with SF-36 scores. However, there was no significant relationship between difference in quadriceps strength, swelling, and quality of life. Previous studies have showed that the sensing system in the human body was more sensitive for the pain feeling and stereoscopic perception [15, 17]. Thus, the differences in ROM and pain could be more perceptible in comparison to quad strength and swelling for patients.

The present study is the first to investigate the question of why interlimb asymmetry occurs and how to improve recovery after SBTKA. The practical implication of this research lies in the identification of risk factors for patients with significantly asymmetrical recovery. This can help medical teams provide better preoperative education and give at-risk patients a realistic expectation of unsynchronized recovery between limbs, which would help to avoid unnecessary postoperative consultation. Secondly, precise knowledge of the natural history of interlimb difference in clinical outcomes following TKA is of fundamental importance to prevent unsatisfactory results. Meanwhile, these data can help physical therapists and surgeons design more scientific and reasonable exercise protocols to reduce the extent of interlimb differences. Specific treatments targeted at different limbs to balance between-limb asymmetry are necessary.

Limitations in this study should be acknowledged. Definition of “asymmetry” in this study was based on 20% interlimb difference in active ROM of knee. The asymmetry of our definition can affect the present conclusions. Secondly, self-reported questionnaires (VAS pain scores and SF-36) were used to assess interlimb difference in pain and quality of life pre- and postoperatively. Self-report data leads to less persuasive conclusions due to recall bias and subjectivity of measurement. Lastly, patients were required to describe pain of each knee to the best of their ability. There were different degrees of potential bias resulting from patients’ ability to distinguish sensations in each knee. Limitations mentioned above open the door for future research and improving these aspects may deepen related conclusions.

## Conclusion

To our knowledge, this is the first study to compare between-limb asymmetry after SBTKA and to detect potential risk factors. Our results demonstrated that significant differences exist between limbs in active ROM, pain level, and quadriceps strength. Significant relationships between interlimb differences in active ROM, pain scores, and quality of life were also determined. Identifying high-risk patients for asymmetry potentially helps to explain between-limb asymmetry for surgeons and physical therapists, to avoid unnecessary medical expense,and to decrease patient dissatisfaction. Further research is needed in this area to study whether individuals may benefit from treatments targeted at different limbs to balance between-limb asymmetry after surgery.

## References

[1] Ivory JP, Simpson AH, Toogood GJ: Bilateral knee replacements: simultaneous or staged? J R Coll Surg Edinb 38:105, 1993 8478827

[2] Ritter M, Mamlin LA, Melfi CA, Katz BP, Freund DA, Arthur DS: Outcome implication for the timing of bilateral total knee arthroplasties. Clin Orthop Relat Res 1997;345:99–105. 9418626

[3] Huotari K, Lyytikäinen O, Seitsalo S: Patient outcomes after simultaneous bilateral total hip and knee joint replacements. J Hosp Infect. 2007 Mar;65(3):219–25. 17275961 10.1016/j.jhin.2006.10.018

[4] Kim YH, Choi YW, Kim JS: Simultaneous bilateral, sequential total knee replacement is as safe as unilateral total knee replacement. J Bone Joint Surg 2009;91B:64–8. 10.1302/0301-620X.91B1.21320 19092006

[5] Worland RL, Jessup DE, Clelland C: Simultaneous bilateral total knee replacement versus unilateral replacement. Am J Orthop 1996;25:292–5. 8728366

[6] Noble J., Goodall J.R., D.J.: Simultaneous Bilateral Total Knee Replacement: A persistent controversy. The Knee 16 (2009) 420–426 10.1016/j.knee.2009.04.009 19464899

[7] Jankiewicz JJ, Sculco TP, Ranawat CS, Behr C, Tarrentino S: One-stage versus 2-stage bilateral total knee arthroplasty. Clin Orthop Relat Res 1994;309:94–101. 7994981

[8] McLaughlin TP, Fisher RL: Bilateral total knee arthroplas ties. Comparison of simultaneous(two-team), sequential, and staged knee replacements. Clin Orthop Relat Res 1985;199:220–225. 4042482

[9] Gong L, Chen H.N. Descriptive analysis of the cost-effectiveness of depressed patients undergoing total knee arthroplasty: an economic decision analysis. J Orthop Sci. 2014 9;19(5):820–6. 10.1007/s00776-014-0599-y 24996623

[10] Cohen RG, Forrest CJ, Benjamin JB: Safety and efficacy of bilateral total knee arthroplasty. J Arthroplasty 1997;12: 497–502. 9268788 10.1016/s0883-5403(97)90171-6

[11] Lane GJ, Hozack WJ, Shah S: Simultaneous bilateral versus unilateral total knee arthroplasty. Outcomes analysis. Clin Orthop Relat Res 1997;345:106–112. 9418627

[12] Parvizi J, Sullivan TA, Trousdale RT, Lewallen DG: Thirty day mortality after total knee arthroplasty. J Bone Joint Surg Am 2001;83-A:1157–1161. 11507123 10.2106/00004623-200108000-00004

[13] Trojani C., Bugnas B., Blay M., Carles M., Boileau P.: Bilateral total knee arthroplasty in a one-stage surgical procedure. Orthopaedics & Traumatology: Surgery & Research (2012) 98, 857–862 10.1016/j.otsr.2012.08.003 23146285

[14] Rossi MD, Brown LE, Whitehurst M, Charni C, Hankins J, Taylor CL: Comparison of knee extensor strength between limbs in individuals with bilateral total knee placement. Arch Phys Med Rehabil. 2002;83(4):523–526. 11932855 10.1053/apmr.2002.30935

[15] Rossi MD, Brown LE, Whitehurst M: Knee extensor and flexor torque characteristics before and after unilateral total knee arthroplasty. Am J Phys Med Rehabil. 2006;85(9):737–746. 16924186 10.1097/01.phm.0000233178.22621.a5

[16] Berman AT, Zarro VJ, Bosacco SJ, Israelite C: Quantitative gait analysis after unilateral or bilateral total knee replacement. J Bone Joint Surg Am. 1987;69(9):1340–1345. 3440793

[17] Brown TE, Diduch DR, Moskal JT: Component size asymmetry in bilateral total knee arthroplasty. Am J Knee Surg. 2001 Spring;14(2):81–4. 11401174

[18] Capeci CM, Brown EC, Scuderi GR, Scott WN: Component asymmetry in simultaneous bilateral total knee arthroplasty. J Arthroplasty. 2006 8;21(5):749–53. 16877164 10.1016/j.arth.2005.09.010

[19] Reddy VG, Mootha AK, Thayi C, Kantesaria P, Kumar RV, Reddy D. Are both the knees of the same size? Analysis of component asymmetry in 289 bilateral knee arthroplasties: Indian J Orthop. 2011 5;45(3):251–4. 10.4103/0019-5413.80044 21559105 PMC3087227

[20] Stubbs G, Pryke SE, Tewari S: Safety and cost benefits of bilateral total knee replacement in an acute hospital. Aust N Z J Surg 2005;75:739–746. 10.1111/j.1445-2197.2005.03516.x 16173984

[21] Duivenvoorden T, Vissers MM, Verhaar JA, Busschbach JJ, Gosens T, Bloem RM et al: Anxiety and depressive symptoms before and after total hip and knee arthroplasty: a prospective multicentre study. Osteoarthritis Cartilage. 2013 12;21(12):1834–40. 10.1016/j.joca.2013.08.022 24012622

[22] Noiseux NO, Callaghan JJ, Clark CR, Zimmerman MB, Sluka KA, Rakel BA.Preoperative predictors of pain following total knee arthroplasty.J Arthroplasty. 2014 7;29(7):1383–7. 10.1016/j.arth.2014.01.034 24630598 PMC4065835

[23] Forsythe ME, Dunbar MJ, Hennigar AW, Sullivan MJ, Gross M. Prospective relation between catastrophizing and residual pain following knee arthroplasty: two-year follow-up. Pain Res Manag. 2008 Jul-Aug;13(4):335–41 18719716 10.1155/2008/730951 PMC2671320

[24] Ali A, Sundberg M, Robertsson O, Dahlberg LE, Thorstensson CA, Redlund-Johnell I: Dissatisfied patients after total knee arthroplasty: a registry study involving 114 patients with 8–13 years of followup. Acta Orthop. 2014 6;85(3):229–33. 10.3109/17453674.2014.916487 24786904 PMC4062787

